# (*E*)-3-[4-(Pent­yloxy)phen­yl]-1-phenyl­prop-2-en-1-one

**DOI:** 10.1107/S1600536809016754

**Published:** 2009-05-14

**Authors:** Asghar Abbas, M. Khawar Rauf, Michael Bolte, Aurangzeb Hasan

**Affiliations:** a Department of Chemistry, Quaid-i-Azam University Islamabad, 45320-Pakistan; b Institut für Anorganische Chemie, J. W. Goethe-Universität Frankfurt, Max-von-Laue-Str. 7, 60438 Frankfurt/Main, Germany

## Abstract

The title compound, C_20_H_22_O_2_, crystallizes with two independent mol­ecules in the asymmetric unit. In each mol­ecule, all the non-H atoms lie in a common plane (r.m.s. deviations of 0.098 and 0.079 Å). There is a π–π stacking inter­action in the crystal structure. The central aromatic rings of the two mol­ecules, which are stacked head-to-tail one above the other, are separated by centroid-to-centroid distances of 3.872 (13) and 3.999 (10) Å.

## Related literature

For background information on chalcones and their properties, see: Achanta *et al.* (2006 ▶); Zhang *et al.* (2009 ▶); Tran *et al.* (2009 ▶); Yagura *et al.* (2008 ▶); Sarissky *et al.* (2008 ▶); Tang *et al.* (2008 ▶); Srivastava *et al.* (2008 ▶); For bond-length data, see: Allen *et al.* (1987 ▶). For related structures, see: Rosli *et al.* (2006 ▶); Harrison *et al.* (2006 ▶). For the synthesis, see: Wattanasin & Murphy (1980 ▶).
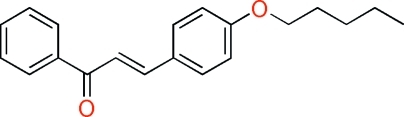


## Experimental

### 

#### Crystal data

C_20_H_22_O_2_
*M* *_r_* = 294.38
Monoclinic, 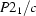

*a* = 7.4881 (4) Å
*b* = 21.3067 (11) Å
*c* = 20.8328 (11) Å
β = 93.974 (4)°
*V* = 3315.8 (3) Å^3^
*Z* = 8
Mo *K*α radiation
μ = 0.07 mm^−1^
*T* = 173 K
0.38 × 0.22 × 0.22 mm

#### Data collection

Stoe IPDS II two-circle-diffractometer
Absorption correction: none
25934 measured reflections
5819 independent reflections
3319 reflections with *I* > 2σ(*I*)
*R*_int_ = 0.086

#### Refinement

*R*[*F* ^2^ > 2σ(*F* ^2^)] = 0.044
*wR*(*F* ^2^) = 0.101
*S* = 0.82
5819 reflections
398 parameters
H-atom parameters constrained
Δρ_max_ = 0.15 e Å^−3^
Δρ_min_ = −0.19 e Å^−3^

### 

Data collection: *X-AREA* (Stoe & Cie, 2001 ▶); cell refinement: *X-AREA*; data reduction: *X-AREA*; program(s) used to solve structure: *SHELXS97* (Sheldrick, 2008 ▶); program(s) used to refine structure: *SHELXL97* (Sheldrick, 2008 ▶); molecular graphics: *XP* in *SHELXTL-Plus* (Sheldrick, 2008 ▶); software used to prepare material for publication: *SHELXL97*.

## Supplementary Material

Crystal structure: contains datablocks I, global. DOI: 10.1107/S1600536809016754/su2111sup1.cif

Structure factors: contains datablocks I. DOI: 10.1107/S1600536809016754/su2111Isup2.hkl

Additional supplementary materials:  crystallographic information; 3D view; checkCIF report

## Figures and Tables

**Table 1:** Selected torsion angles (°)

C11—C1—C2—C3	−177.21 (17)
C2—C1—C11—C12	−13.4 (3)
C2—C1—C11—C16	167.85 (17)
C11*A*—C1*A*—C2*A*—C3*A*	−170.05 (18)
C2*A*—C1*A*—C11*A*—C12*A*	−9.5 (3)
C2*A*—C1*A*—C11*A*—C16*A*	171.52 (17)

**Fig. 1.**
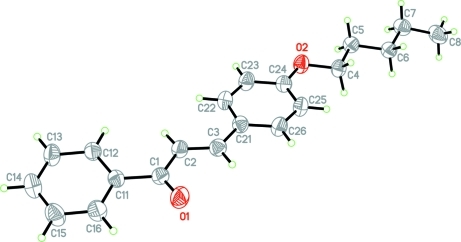
a. Molecular structure of molecule A of the title compound in the asymmetric unit. Displacement ellipsoids are drawn at the 50% probability level.

**Fig. 2.**
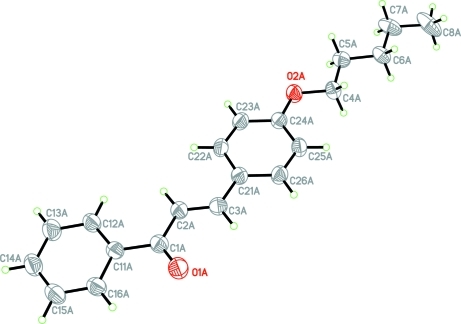
b. Molecular structure of the molecule B of the title compound in the asymmetric unit. Displacement ellipsoids are drawn at the 50% probability level.

**Fig. 3.**
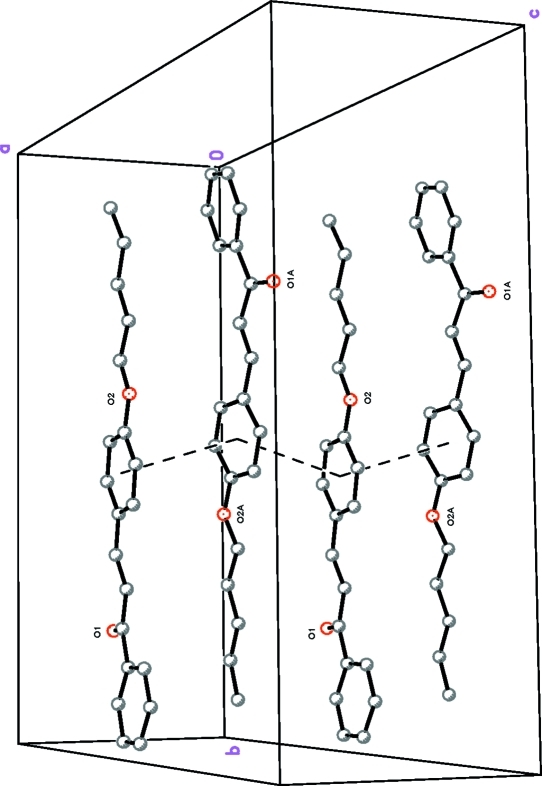
Crystal packing of the title compound with the π-π-stacking interactions shown as dashed lines. The hydrogen atoms have been removed for clarity.

## supplementary crystallographic information

### Comment

Benzylideneacetophenones (α, β-Unsaturated ketones) comprise a class of synthetic and naturally occurring compounds belonging to the flavonoid family of compounds, commonly known as *"chalcones"*. Chalcones and their derivatives are used as precursors for the synthesis of a variety of bioactive organic compounds including heterocyclic compounds. Chalcone analogues have been reported to exhibits potent anticancer activity through inhibition of the proteasome (Achanta *et al.*, 2006), tyrosinase (Zhang *et al.*, 2009) and prostaglandin E-2 (Tran*et al.*, 2009). Naringenin chalcone, the aglycone of isosalipurposide (Yagura *et al.*, 2008) have strong anti-proliferative activity. Some synthetic (Sarissky *et al.*, 2008) and naturally occurring chalcones (Tang *et al.*, 2008) have shown good anticancer activity against Ben-Men-1 human benign meningioma cell line and bladder cancer. Oxygenated chalcones and bischalcones (Srivastava *et al.*,2008) are a new class of inhibitors of DNA topoisomerase-II of malarial parasites. Here, we report on the crystal structure of the title compound, which was prepared and used as a precursor for the synthesis of heterocyclic compounds.

The molecular structue of the title compound is shown in Fig. 1. The geometrical parameters are normal (Allen *et al.*, 1987) and consistent with those of recently reported chalcone derivatives (Rosli *et al.*, 2006). The asymmetric unit consists of two independent conformers distinctly twisted about the C11—C1 / C11A—C1A and the C1—C2 / C1A—C2A bonds (Table 1), as was also observed for 2-bromo-1-chlorophenyl-3-(4-methoxyphenyl)-2-propen-1-one (Harrison *et al.*, 2006). The dihedral angle between the benzene ring mean planes (C11–C16) and (C21–C26), and (C11A–C16A) and (C21A–C26A) are 12.33 (4) and 7.63 (2)°, respectively. Atoms C1 and O1 deviate from the mean plane (C11–C16) by 0.035 (3) and 0.291 (3) Å, respectively. While atoms C1A and O1A deviated from the mean plane (C11A–C16A) by 0.033 (3) and 0.209 (3) Å, respectively.

In the crystal structure of the title compound the two independent molecules stack head-to-tail (Fig. 2). The central aromatic rings of the two molecules are separated by centroid-to-centroid distances of ca. 3.872 and 3.999 Å.

### Experimental

The title compound was synthesized by base catalyzed Claisen–Schmidt condensation reaction (Wattanasin *et al.*, 1980). A mixture of acetophenone (1.20 g, 0.01 mol) and 4-(pentyloxy)benzaldehyde (1.92 g, 0.01 mol) was dissolved in ethanol (50 ml) and then 20 ml of an aqueous solution of potassium hydroxide (5%) was added. The mixture was stirred for 3–4 hr, neutralized with dilute HCl and left to stand for 12 hr. The resulting crude solid mass was collected by filtration and recrystallized from ethanol, yielding crystals of the title compound. Full spectroscopic and physical characterization will be reported elsewhere.

### Refinement

Hydrogen atoms were located in a difference Fourier map but the were included in calculated positions [C-H = 0.95 - 0.99 Å] and refined as riding [U_iso_(H) = 1.2 or 1.5U_eq_(parent C-atom)]

### Crystal data

**Table:**

C_20_H_22_O_2_	*F*(000) = 1264
*M**_r_* = 294.38	*D*_x_ = 1.179 Mg m^−^^3^
Monoclinic, *P*2_1_/*c*	Mo *K*α radiation, λ = 0.71073 Å
Hall symbol: -P 2ybc	Cell parameters from 10828 reflections
*a* = 7.4881 (4) Å	θ = 2.2–25.4°
*b* = 21.3067 (11) Å	µ = 0.07 mm^−^^1^
*c* = 20.8328 (11) Å	*T* = 173 K
β = 93.974 (4)°	Needle, light yellow
*V* = 3315.8 (3) Å^3^	0.38 × 0.22 × 0.22 mm
*Z* = 8	

### Data collection

**Table:**

STOE IPDS II two-circle-diffractometer	3319 reflections with *I* > 2σ(*I*)
Radiation source: fine-focus sealed tube	*R*_int_ = 0.086
graphite	θ_max_ = 25.0°, θ_min_ = 2.2°
ω scans	*h* = −8→8
25934 measured reflections	*k* = −25→25
5819 independent reflections	*l* = −24→24

### Refinement

**Table:**

Refinement on *F*^2^	Secondary atom site location: difference Fourier map
Least-squares matrix: full	Hydrogen site location: inferred from neighbouring sites
*R*[*F*^2^ > 2σ(*F*^2^)] = 0.044	H-atom parameters constrained
*wR*(*F*^2^) = 0.101	*w* = 1/[σ^2^(*F*_o_^2^) + (0.0461*P*)^2^] where *P* = (*F*_o_^2^ + 2*F*_c_^2^)/3
*S* = 0.82	(Δ/σ)_max_ < 0.001
5819 reflections	Δρ_max_ = 0.15 e Å^−^^3^
398 parameters	Δρ_min_ = −0.18 e Å^−^^3^
0 restraints	Extinction correction: *SHELXL*, Fc^*^=kFc[1+0.001xFc^2^λ^3^/sin(2θ)]^-1/4^
Primary atom site location: structure-invariant direct methods	Extinction coefficient: 0.0076 (8)

### Special details

**Table:**

Geometry. All e.s.d.'s (except the e.s.d. in the dihedral angle between two l.s. planes) are estimated using the full covariance matrix. The cell e.s.d.'s are taken into account individually in the estimation of e.s.d.'s in distances, angles and torsion angles; correlations between e.s.d.'s in cell parameters are only used when they are defined by crystal symmetry. An approximate (isotropic) treatment of cell e.s.d.'s is used for estimating e.s.d.'s involving l.s. planes.
Refinement. Refinement of *F*^2^ against ALL reflections. The weighted *R*-factor *wR* and goodness of fit *S* are based on *F*^2^, conventional *R*-factors *R* are based on *F*, with *F* set to zero for negative *F*^2^. The threshold expression of *F*^2^ > σ(*F*^2^) is used only for calculating *R*-factors(gt) *etc*. and is not relevant to the choice of reflections for refinement. *R*-factors based on *F*^2^ are statistically about twice as large as those based on *F*, and *R*- factors based on ALL data will be even larger.

### Fractional atomic coordinates and isotropic or equivalent isotropic displacement parameters (Å^2^)

**Table:**

	*x*	*y*	*z*	*U*_iso_*/*U*_eq_	
O1	0.3753 (2)	0.28357 (6)	0.50789 (6)	0.0563 (4)	
O2	0.36921 (18)	0.62946 (6)	0.31330 (6)	0.0438 (3)	
C1	0.4030 (2)	0.27970 (9)	0.45038 (8)	0.0374 (4)	
C2	0.4045 (2)	0.33580 (9)	0.40938 (8)	0.0377 (4)	
H2	0.4199	0.3314	0.3647	0.045*	
C3	0.3843 (2)	0.39303 (9)	0.43424 (9)	0.0391 (4)	
H3	0.3695	0.3943	0.4791	0.047*	
C4	0.3530 (3)	0.68540 (8)	0.35105 (8)	0.0404 (5)	
H4A	0.4567	0.6891	0.3830	0.049*	
H4B	0.2425	0.6836	0.3745	0.049*	
C5	0.3465 (3)	0.74114 (9)	0.30660 (8)	0.0410 (5)	
H5A	0.2375	0.7387	0.2769	0.049*	
H5B	0.4519	0.7403	0.2804	0.049*	
C6	0.3451 (3)	0.80230 (9)	0.34415 (8)	0.0407 (5)	
H6A	0.2424	0.8018	0.3716	0.049*	
H6B	0.4558	0.8048	0.3729	0.049*	
C7	0.3323 (3)	0.86063 (9)	0.30226 (9)	0.0485 (5)	
H7A	0.4293	0.8596	0.2723	0.058*	
H7B	0.2168	0.8600	0.2761	0.058*	
C8	0.3457 (3)	0.92128 (10)	0.34037 (12)	0.0625 (6)	
H8A	0.3372	0.9570	0.3107	0.094*	
H8B	0.4608	0.9227	0.3658	0.094*	
H8C	0.2480	0.9233	0.3692	0.094*	
C11	0.4342 (2)	0.21639 (9)	0.42220 (8)	0.0344 (4)	
C12	0.4295 (2)	0.20512 (9)	0.35622 (8)	0.0400 (4)	
H12	0.4078	0.2388	0.3269	0.048*	
C13	0.4562 (3)	0.14507 (9)	0.33307 (9)	0.0481 (5)	
H13	0.4541	0.1379	0.2880	0.058*	
C14	0.4859 (3)	0.09584 (10)	0.37528 (10)	0.0527 (5)	
H14	0.5021	0.0546	0.3593	0.063*	
C15	0.4920 (3)	0.10636 (10)	0.44094 (10)	0.0544 (6)	
H15	0.5140	0.0725	0.4701	0.065*	
C16	0.4661 (3)	0.16618 (9)	0.46385 (9)	0.0463 (5)	
H16	0.4701	0.1731	0.5090	0.056*	
C21	0.3818 (2)	0.45381 (9)	0.40194 (8)	0.0366 (4)	
C22	0.4064 (2)	0.46178 (9)	0.33614 (8)	0.0419 (5)	
H22	0.4264	0.4261	0.3103	0.050*	
C23	0.4021 (3)	0.52028 (9)	0.30860 (9)	0.0438 (5)	
H23	0.4190	0.5246	0.2640	0.053*	
C24	0.3730 (2)	0.57350 (9)	0.34540 (8)	0.0373 (4)	
C25	0.3491 (3)	0.56698 (9)	0.41056 (8)	0.0422 (5)	
H25	0.3308	0.6029	0.4363	0.051*	
C26	0.3522 (3)	0.50777 (9)	0.43742 (9)	0.0431 (5)	
H26	0.3334	0.5036	0.4818	0.052*	
O1A	0.8034 (2)	0.81245 (6)	0.24815 (6)	0.0549 (4)	
O2A	0.90510 (18)	0.45190 (6)	0.41506 (5)	0.0432 (3)	
C1A	0.8272 (2)	0.80873 (9)	0.30728 (8)	0.0386 (4)	
C2A	0.8414 (2)	0.74769 (9)	0.33972 (8)	0.0383 (4)	
H2A	0.8414	0.7464	0.3853	0.046*	
C3A	0.8545 (2)	0.69393 (9)	0.30771 (8)	0.0386 (4)	
H3A	0.8536	0.6969	0.2622	0.046*	
C4A	0.9039 (3)	0.39658 (8)	0.37602 (8)	0.0407 (4)	
H4A1	1.0081	0.3967	0.3493	0.049*	
H4A2	0.7933	0.3951	0.3471	0.049*	
C5A	0.9124 (3)	0.34046 (9)	0.42018 (9)	0.0422 (5)	
H5A1	0.8057	0.3402	0.4457	0.051*	
H5A2	1.0198	0.3438	0.4505	0.051*	
C6A	0.9201 (3)	0.27920 (8)	0.38292 (9)	0.0448 (5)	
H6A1	0.8104	0.2755	0.3538	0.054*	
H6A2	1.0237	0.2806	0.3559	0.054*	
C7A	0.9362 (3)	0.22144 (10)	0.42529 (11)	0.0575 (6)	
H7A1	0.8292	0.2185	0.4505	0.069*	
H7A2	1.0424	0.2259	0.4560	0.069*	
C8A	0.9535 (3)	0.16114 (10)	0.38681 (14)	0.0798 (8)	
H8A1	0.9630	0.1253	0.4163	0.120*	
H8A2	1.0609	0.1633	0.3626	0.120*	
H8A3	0.8477	0.1561	0.3568	0.120*	
C11A	0.8432 (2)	0.86778 (9)	0.34646 (8)	0.0364 (4)	
C12A	0.8455 (3)	0.86841 (9)	0.41317 (8)	0.0441 (5)	
H12A	0.8404	0.8300	0.4361	0.053*	
C13A	0.8553 (3)	0.92462 (10)	0.44644 (10)	0.0533 (5)	
H13A	0.8548	0.9246	0.4920	0.064*	
C14A	0.8656 (3)	0.98053 (10)	0.41389 (10)	0.0525 (5)	
H14A	0.8725	1.0190	0.4370	0.063*	
C15A	0.8661 (3)	0.98060 (9)	0.34781 (10)	0.0512 (5)	
H15A	0.8751	1.0192	0.3254	0.061*	
C16A	0.8533 (3)	0.92468 (9)	0.31398 (9)	0.0437 (5)	
H16A	0.8514	0.9251	0.2683	0.052*	
C21A	0.8699 (2)	0.63097 (9)	0.33500 (8)	0.0367 (4)	
C22A	0.8770 (3)	0.62003 (9)	0.40134 (8)	0.0412 (5)	
H22A	0.8715	0.6546	0.4299	0.049*	
C23A	0.8918 (3)	0.56028 (9)	0.42609 (9)	0.0431 (5)	
H23A	0.8994	0.5542	0.4714	0.052*	
C24A	0.8957 (2)	0.50891 (8)	0.38534 (8)	0.0361 (4)	
C25A	0.8902 (2)	0.51804 (9)	0.31898 (8)	0.0405 (5)	
H25A	0.8943	0.4833	0.2906	0.049*	
C26A	0.8787 (3)	0.57867 (9)	0.29517 (8)	0.0420 (5)	
H26A	0.8766	0.5849	0.2500	0.050*	

### Atomic displacement parameters (Å^2^)

**Table:**

	*U*^11^	*U*^22^	*U*^33^	*U*^12^	*U*^13^	*U*^23^
O1	0.0865 (11)	0.0481 (9)	0.0348 (7)	0.0088 (8)	0.0074 (7)	−0.0041 (6)
O2	0.0578 (8)	0.0340 (8)	0.0403 (7)	−0.0030 (6)	0.0070 (6)	−0.0019 (6)
C1	0.0362 (10)	0.0384 (11)	0.0372 (10)	0.0006 (8)	0.0002 (8)	−0.0023 (8)
C2	0.0400 (11)	0.0369 (11)	0.0365 (9)	0.0005 (9)	0.0039 (8)	−0.0026 (8)
C3	0.0373 (10)	0.0402 (12)	0.0398 (10)	−0.0013 (9)	0.0016 (8)	−0.0026 (8)
C4	0.0469 (11)	0.0327 (11)	0.0419 (10)	−0.0040 (9)	0.0052 (8)	−0.0031 (8)
C5	0.0434 (11)	0.0382 (11)	0.0415 (10)	−0.0026 (9)	0.0034 (8)	0.0014 (8)
C6	0.0416 (11)	0.0379 (11)	0.0428 (10)	−0.0014 (9)	0.0033 (8)	0.0011 (8)
C7	0.0478 (12)	0.0419 (12)	0.0556 (12)	−0.0010 (10)	0.0019 (9)	0.0054 (9)
C8	0.0582 (14)	0.0398 (13)	0.0881 (16)	0.0016 (11)	−0.0051 (12)	0.0027 (12)
C11	0.0310 (9)	0.0323 (10)	0.0401 (9)	0.0001 (8)	0.0037 (7)	−0.0001 (8)
C12	0.0456 (11)	0.0365 (11)	0.0380 (9)	0.0028 (9)	0.0036 (8)	−0.0008 (8)
C13	0.0592 (13)	0.0392 (12)	0.0470 (11)	0.0030 (10)	0.0114 (9)	−0.0082 (9)
C14	0.0604 (14)	0.0357 (12)	0.0638 (13)	0.0038 (10)	0.0168 (10)	−0.0028 (10)
C15	0.0715 (15)	0.0389 (12)	0.0540 (12)	0.0060 (11)	0.0131 (11)	0.0108 (10)
C16	0.0553 (12)	0.0422 (12)	0.0425 (10)	0.0025 (10)	0.0104 (9)	0.0017 (9)
C21	0.0343 (10)	0.0344 (11)	0.0409 (10)	−0.0019 (8)	0.0031 (8)	−0.0026 (8)
C22	0.0464 (11)	0.0390 (12)	0.0410 (10)	−0.0002 (9)	0.0079 (8)	−0.0077 (9)
C23	0.0549 (12)	0.0406 (12)	0.0369 (10)	−0.0022 (10)	0.0099 (9)	−0.0036 (9)
C24	0.0380 (10)	0.0343 (11)	0.0400 (10)	−0.0042 (8)	0.0046 (8)	0.0012 (8)
C25	0.0527 (12)	0.0341 (11)	0.0405 (10)	−0.0031 (9)	0.0080 (8)	−0.0071 (8)
C26	0.0545 (12)	0.0387 (12)	0.0368 (10)	−0.0040 (10)	0.0077 (9)	−0.0038 (8)
O1A	0.0860 (11)	0.0418 (8)	0.0360 (7)	0.0002 (8)	−0.0008 (7)	0.0018 (6)
O2A	0.0588 (9)	0.0313 (8)	0.0397 (7)	0.0033 (6)	0.0043 (6)	0.0012 (6)
C1A	0.0390 (11)	0.0364 (11)	0.0404 (10)	0.0007 (9)	0.0022 (8)	0.0015 (8)
C2A	0.0435 (11)	0.0351 (11)	0.0364 (9)	0.0001 (9)	0.0035 (8)	0.0001 (8)
C3A	0.0416 (11)	0.0381 (11)	0.0360 (9)	0.0008 (9)	0.0015 (8)	0.0020 (8)
C4A	0.0464 (11)	0.0333 (11)	0.0418 (10)	0.0007 (9)	−0.0011 (8)	−0.0022 (8)
C5A	0.0406 (11)	0.0402 (12)	0.0460 (11)	−0.0001 (9)	0.0042 (8)	0.0038 (9)
C6A	0.0455 (11)	0.0323 (11)	0.0557 (11)	−0.0029 (9)	−0.0018 (9)	0.0051 (9)
C7A	0.0435 (12)	0.0408 (13)	0.0879 (15)	−0.0026 (10)	0.0012 (11)	0.0214 (11)
C8A	0.0639 (16)	0.0321 (13)	0.140 (2)	−0.0054 (12)	−0.0151 (15)	0.0103 (14)
C11A	0.0342 (10)	0.0334 (11)	0.0416 (10)	−0.0008 (8)	0.0034 (8)	0.0013 (8)
C12A	0.0568 (13)	0.0344 (11)	0.0408 (10)	−0.0011 (10)	0.0012 (9)	0.0023 (8)
C13A	0.0693 (15)	0.0438 (13)	0.0464 (11)	−0.0047 (11)	0.0003 (10)	−0.0061 (10)
C14A	0.0574 (13)	0.0383 (13)	0.0618 (14)	−0.0052 (10)	0.0042 (10)	−0.0089 (10)
C15A	0.0555 (13)	0.0343 (12)	0.0639 (13)	−0.0018 (10)	0.0055 (10)	0.0062 (10)
C16A	0.0493 (12)	0.0364 (11)	0.0453 (11)	−0.0014 (9)	0.0035 (9)	0.0052 (9)
C21A	0.0387 (10)	0.0334 (11)	0.0381 (10)	0.0021 (8)	0.0033 (8)	−0.0004 (8)
C22A	0.0544 (12)	0.0344 (11)	0.0353 (9)	0.0040 (9)	0.0067 (8)	−0.0049 (8)
C23A	0.0582 (13)	0.0377 (12)	0.0343 (9)	0.0056 (9)	0.0098 (8)	0.0011 (8)
C24A	0.0386 (11)	0.0334 (11)	0.0364 (10)	0.0035 (8)	0.0026 (8)	0.0016 (8)
C25A	0.0498 (12)	0.0326 (11)	0.0387 (10)	0.0029 (9)	0.0009 (8)	−0.0051 (8)
C26A	0.0538 (12)	0.0390 (12)	0.0330 (9)	0.0031 (9)	0.0025 (8)	−0.0008 (8)

### Geometric parameters (Å, °)

**Table:**

O1—C1	1.2326 (19)	O1A—C1A	1.235 (2)
O2—C24	1.366 (2)	O2A—C24A	1.363 (2)
O2—C4	1.438 (2)	O2A—C4A	1.432 (2)
C1—C2	1.470 (3)	C1A—C2A	1.466 (3)
C1—C11	1.496 (3)	C1A—C11A	1.500 (3)
C2—C3	1.338 (3)	C2A—C3A	1.332 (2)
C2—H2	0.9500	C2A—H2A	0.9500
C3—C21	1.459 (3)	C3A—C21A	1.459 (3)
C3—H3	0.9500	C3A—H3A	0.9500
C4—C5	1.505 (2)	C4A—C5A	1.507 (2)
C4—H4A	0.9900	C4A—H4A1	0.9900
C4—H4B	0.9900	C4A—H4A2	0.9900
C5—C6	1.520 (3)	C5A—C6A	1.522 (3)
C5—H5A	0.9900	C5A—H5A1	0.9900
C5—H5B	0.9900	C5A—H5A2	0.9900
C6—C7	1.518 (3)	C6A—C7A	1.515 (3)
C6—H6A	0.9900	C6A—H6A1	0.9900
C6—H6B	0.9900	C6A—H6A2	0.9900
C7—C8	1.516 (3)	C7A—C8A	1.525 (3)
C7—H7A	0.9900	C7A—H7A1	0.9900
C7—H7B	0.9900	C7A—H7A2	0.9900
C8—H8A	0.9800	C8A—H8A1	0.9800
C8—H8B	0.9800	C8A—H8A2	0.9800
C8—H8C	0.9800	C8A—H8A3	0.9800
C11—C16	1.388 (3)	C11A—C12A	1.389 (2)
C11—C12	1.393 (2)	C11A—C16A	1.393 (3)
C12—C13	1.387 (3)	C12A—C13A	1.383 (3)
C12—H12	0.9500	C12A—H12A	0.9500
C13—C14	1.377 (3)	C13A—C14A	1.376 (3)
C13—H13	0.9500	C13A—H13A	0.9500
C14—C15	1.384 (3)	C14A—C15A	1.377 (3)
C14—H14	0.9500	C14A—H14A	0.9500
C15—C16	1.379 (3)	C15A—C16A	1.384 (3)
C15—H15	0.9500	C15A—H15A	0.9500
C16—H16	0.9500	C16A—H16A	0.9500
C21—C26	1.393 (3)	C21A—C26A	1.394 (3)
C21—C22	1.406 (2)	C21A—C22A	1.399 (2)
C22—C23	1.372 (3)	C22A—C23A	1.375 (3)
C22—H22	0.9500	C22A—H22A	0.9500
C23—C24	1.394 (3)	C23A—C24A	1.387 (2)
C23—H23	0.9500	C23A—H23A	0.9500
C24—C25	1.388 (2)	C24A—C25A	1.394 (2)
C25—C26	1.380 (3)	C25A—C26A	1.384 (3)
C25—H25	0.9500	C25A—H25A	0.9500
C26—H26	0.9500	C26A—H26A	0.9500
			
C24—O2—C4	117.09 (13)	C24A—O2A—C4A	118.52 (13)
O1—C1—C2	121.27 (17)	O1A—C1A—C2A	121.18 (17)
O1—C1—C11	118.89 (16)	O1A—C1A—C11A	119.30 (17)
C2—C1—C11	119.83 (15)	C2A—C1A—C11A	119.52 (16)
C3—C2—C1	120.71 (16)	C3A—C2A—C1A	122.50 (16)
C3—C2—H2	119.6	C3A—C2A—H2A	118.8
C1—C2—H2	119.6	C1A—C2A—H2A	118.8
C2—C3—C21	128.96 (17)	C2A—C3A—C21A	127.02 (17)
C2—C3—H3	115.5	C2A—C3A—H3A	116.5
C21—C3—H3	115.5	C21A—C3A—H3A	116.5
O2—C4—C5	108.55 (14)	O2A—C4A—C5A	107.93 (14)
O2—C4—H4A	110.0	O2A—C4A—H4A1	110.1
C5—C4—H4A	110.0	C5A—C4A—H4A1	110.1
O2—C4—H4B	110.0	O2A—C4A—H4A2	110.1
C5—C4—H4B	110.0	C5A—C4A—H4A2	110.1
H4A—C4—H4B	108.4	H4A1—C4A—H4A2	108.4
C4—C5—C6	111.17 (15)	C4A—C5A—C6A	111.76 (15)
C4—C5—H5A	109.4	C4A—C5A—H5A1	109.3
C6—C5—H5A	109.4	C6A—C5A—H5A1	109.3
C4—C5—H5B	109.4	C4A—C5A—H5A2	109.3
C6—C5—H5B	109.4	C6A—C5A—H5A2	109.3
H5A—C5—H5B	108.0	H5A1—C5A—H5A2	107.9
C7—C6—C5	114.12 (15)	C7A—C6A—C5A	113.83 (16)
C7—C6—H6A	108.7	C7A—C6A—H6A1	108.8
C5—C6—H6A	108.7	C5A—C6A—H6A1	108.8
C7—C6—H6B	108.7	C7A—C6A—H6A2	108.8
C5—C6—H6B	108.7	C5A—C6A—H6A2	108.8
H6A—C6—H6B	107.6	H6A1—C6A—H6A2	107.7
C8—C7—C6	113.43 (17)	C6A—C7A—C8A	112.62 (19)
C8—C7—H7A	108.9	C6A—C7A—H7A1	109.1
C6—C7—H7A	108.9	C8A—C7A—H7A1	109.1
C8—C7—H7B	108.9	C6A—C7A—H7A2	109.1
C6—C7—H7B	108.9	C8A—C7A—H7A2	109.1
H7A—C7—H7B	107.7	H7A1—C7A—H7A2	107.8
C7—C8—H8A	109.5	C7A—C8A—H8A1	109.5
C7—C8—H8B	109.5	C7A—C8A—H8A2	109.5
H8A—C8—H8B	109.5	H8A1—C8A—H8A2	109.5
C7—C8—H8C	109.5	C7A—C8A—H8A3	109.5
H8A—C8—H8C	109.5	H8A1—C8A—H8A3	109.5
H8B—C8—H8C	109.5	H8A2—C8A—H8A3	109.5
C16—C11—C12	118.38 (17)	C12A—C11A—C16A	118.70 (18)
C16—C11—C1	118.33 (15)	C12A—C11A—C1A	123.21 (17)
C12—C11—C1	123.28 (16)	C16A—C11A—C1A	118.08 (16)
C13—C12—C11	120.51 (18)	C13A—C12A—C11A	120.43 (18)
C13—C12—H12	119.7	C13A—C12A—H12A	119.8
C11—C12—H12	119.7	C11A—C12A—H12A	119.8
C14—C13—C12	120.10 (18)	C14A—C13A—C12A	120.37 (19)
C14—C13—H13	119.9	C14A—C13A—H13A	119.8
C12—C13—H13	119.9	C12A—C13A—H13A	119.8
C13—C14—C15	120.05 (19)	C13A—C14A—C15A	119.9 (2)
C13—C14—H14	120.0	C13A—C14A—H14A	120.1
C15—C14—H14	120.0	C15A—C14A—H14A	120.1
C16—C15—C14	119.72 (19)	C14A—C15A—C16A	120.21 (19)
C16—C15—H15	120.1	C14A—C15A—H15A	119.9
C14—C15—H15	120.1	C16A—C15A—H15A	119.9
C15—C16—C11	121.22 (18)	C15A—C16A—C11A	120.40 (18)
C15—C16—H16	119.4	C15A—C16A—H16A	119.8
C11—C16—H16	119.4	C11A—C16A—H16A	119.8
C26—C21—C22	117.01 (17)	C26A—C21A—C22A	117.06 (17)
C26—C21—C3	119.03 (16)	C26A—C21A—C3A	120.60 (16)
C22—C21—C3	123.96 (17)	C22A—C21A—C3A	122.34 (16)
C23—C22—C21	121.11 (17)	C23A—C22A—C21A	121.41 (17)
C23—C22—H22	119.4	C23A—C22A—H22A	119.3
C21—C22—H22	119.4	C21A—C22A—H22A	119.3
C22—C23—C24	120.61 (17)	C22A—C23A—C24A	120.38 (17)
C22—C23—H23	119.7	C22A—C23A—H23A	119.8
C24—C23—H23	119.7	C24A—C23A—H23A	119.8
O2—C24—C25	124.47 (17)	O2A—C24A—C23A	115.34 (15)
O2—C24—C23	116.06 (15)	O2A—C24A—C25A	124.88 (16)
C25—C24—C23	119.47 (18)	C23A—C24A—C25A	119.78 (17)
C26—C25—C24	119.22 (17)	C26A—C25A—C24A	118.84 (17)
C26—C25—H25	120.4	C26A—C25A—H25A	120.6
C24—C25—H25	120.4	C24A—C25A—H25A	120.6
C25—C26—C21	122.57 (17)	C25A—C26A—C21A	122.49 (17)
C25—C26—H26	118.7	C25A—C26A—H26A	118.8
C21—C26—H26	118.7	C21A—C26A—H26A	118.8
			
O1—C1—C2—C3	3.2 (3)	O1A—C1A—C2A—C3A	9.5 (3)
C11—C1—C2—C3	−177.21 (17)	C11A—C1A—C2A—C3A	−170.05 (18)
C1—C2—C3—C21	−179.97 (18)	C1A—C2A—C3A—C21A	179.74 (18)
C24—O2—C4—C5	−178.53 (15)	C24A—O2A—C4A—C5A	−179.11 (16)
O2—C4—C5—C6	−175.23 (15)	O2A—C4A—C5A—C6A	−177.50 (16)
C4—C5—C6—C7	−178.06 (17)	C4A—C5A—C6A—C7A	177.72 (17)
C5—C6—C7—C8	−175.46 (18)	C5A—C6A—C7A—C8A	−176.95 (18)
O1—C1—C11—C16	−12.6 (3)	O1A—C1A—C11A—C12A	170.93 (18)
C2—C1—C11—C12	−13.4 (3)	C2A—C1A—C11A—C12A	−9.5 (3)
C2—C1—C11—C16	167.85 (17)	O1A—C1A—C11A—C16A	−8.0 (3)
O1—C1—C11—C12	166.19 (17)	C2A—C1A—C11A—C16A	171.52 (17)
C16—C11—C12—C13	0.0 (3)	C16A—C11A—C12A—C13A	0.8 (3)
C1—C11—C12—C13	−178.79 (18)	C1A—C11A—C12A—C13A	−178.11 (19)
C11—C12—C13—C14	0.7 (3)	C11A—C12A—C13A—C14A	−1.0 (3)
C12—C13—C14—C15	−1.1 (3)	C12A—C13A—C14A—C15A	0.1 (3)
C13—C14—C15—C16	0.8 (3)	C13A—C14A—C15A—C16A	1.0 (3)
C14—C15—C16—C11	−0.2 (3)	C14A—C15A—C16A—C11A	−1.2 (3)
C12—C11—C16—C15	−0.2 (3)	C12A—C11A—C16A—C15A	0.3 (3)
C1—C11—C16—C15	178.60 (18)	C1A—C11A—C16A—C15A	179.26 (18)
C2—C3—C21—C26	177.7 (2)	C2A—C3A—C21A—C26A	178.60 (19)
C2—C3—C21—C22	−1.8 (3)	C2A—C3A—C21A—C22A	−1.5 (3)
C26—C21—C22—C23	0.3 (3)	C26A—C21A—C22A—C23A	−0.1 (3)
C3—C21—C22—C23	179.79 (19)	C3A—C21A—C22A—C23A	−179.92 (18)
C21—C22—C23—C24	0.0 (3)	C21A—C22A—C23A—C24A	−1.6 (3)
C4—O2—C24—C25	5.0 (3)	C4A—O2A—C24A—C23A	178.06 (17)
C4—O2—C24—C23	−175.43 (17)	C4A—O2A—C24A—C25A	−2.0 (3)
C22—C23—C24—O2	−179.33 (17)	C22A—C23A—C24A—O2A	−178.06 (17)
C22—C23—C24—C25	0.3 (3)	C22A—C23A—C24A—C25A	2.0 (3)
O2—C24—C25—C26	178.69 (18)	O2A—C24A—C25A—C26A	179.28 (17)
C23—C24—C25—C26	−0.9 (3)	C23A—C24A—C25A—C26A	−0.8 (3)
C24—C25—C26—C21	1.2 (3)	C24A—C25A—C26A—C21A	−0.9 (3)
C22—C21—C26—C25	−0.9 (3)	C22A—C21A—C26A—C25A	1.3 (3)
C3—C21—C26—C25	179.55 (18)	C3A—C21A—C26A—C25A	−178.83 (18)
